# Overlapping DNA Methylation Dynamics in Mouse Intestinal Cell Differentiation and Early Stages of Malignant Progression

**DOI:** 10.1371/journal.pone.0123263

**Published:** 2015-05-01

**Authors:** Marta Forn, Anna Díez-Villanueva, Anna Merlos-Suárez, Mar Muñoz, Sergi Lois, Elvira Carriò, Mireia Jordà, Anna Bigas, Eduard Batlle, Miguel A. Peinado

**Affiliations:** 1 Institute of Predictive and Personalized Medicine of Cancer (IMPPC) 08916 Badalona, Barcelona, Spain; 2 Institute for Research in Biomedicine (IRB Barcelona) 08028 Barcelona, Spain; 3 Institut Hospital del Mar d’Investigació Mèdica (IMIM) 08003 Barcelona, Spain; 4 Institució Catalana de Recerca i Estudis Avançats (ICREA), Barcelona, Spain; CEA - Institut de Genomique, FRANCE

## Abstract

Mouse models of intestinal crypt cell differentiation and tumorigenesis have been used to characterize the molecular mechanisms underlying both processes. DNA methylation is a key epigenetic mark and plays an important role in cell identity and differentiation programs and cancer. To get insights into the dynamics of cell differentiation and malignant transformation we have compared the DNA methylation profiles along the mouse small intestine crypt and early stages of tumorigenesis. Genome-scale analysis of DNA methylation together with microarray gene expression have been applied to compare intestinal crypt stem cells (*EphB2^high^*), differentiated cells (*EphB2^negative^*), *Apc^Min/+^* adenomas and the corresponding non-tumor adjacent tissue, together with small and large intestine samples and the colon cancer cell line CT26. Compared with late stages, small intestine crypt differentiation and early stages of tumorigenesis display few and relatively small changes in DNA methylation. Hypermethylated loci are largely shared by the two processes and affect the proximities of promoter and enhancer regions, with enrichment in genes associated with the intestinal stem cell signature and the PRC2 complex. The hypermethylation is progressive, with minute levels in differentiated cells, as compared with intestinal stem cells, and reaching full methylation in advanced stages. Hypomethylation shows different signatures in differentiation and cancer and is already present in the non-tumor tissue adjacent to the adenomas in *Apc^Min/+^* mice, but at lower levels than advanced cancers. This study provides a reference framework to decipher the mechanisms driving mouse intestinal tumorigenesis and also the human counterpart.

## Introduction

The intestine is a fascinating organ exposed to very dynamic environmental factors including food, toxins and a complex microbiota. A powerful immunological system developed during breastfeeding and weaning provides protection against a wide variety of harmful agents [1]. Nevertheless, the maintenance of a life-long functionality (basically the capability to absorb nutrients) and resisting the multiple hazards is accomplished through an intensive regeneration of intestinal mucosa [1]. Deregulation of cell proliferation and differentiation processes may lead to malignant transformation and propagation of intestinal cancer stem cells [2]. In the mouse, the intestinal epithelial layer has a turnover of 5 days and is folded forming the crypts [3]. Two distinct pools of Intestinal Stem Cells (ISCs) coexist in the crypt bottom: the quiescent ISCs at +4 position (Paneth cells precursors and crypt restorers after injury) and the rapidly cycling crypt-based columnar cells, which are responsible for crypt homeostasis [4]. The cellular differentiation occurs along the crypt axis and the terminally differentiated cells of the small intestine migrate towards the tips of villi (protrusions into the small intestine lumen). Important methodological advances have been made in crypt’s cells isolation and propagation [3,5,6] allowing the characterization of the expression signatures of the different cellular fractions [2,7,8,9,10,11,12,13,14].

DNA methylation is the main epigenetic modification of DNA and, together with other epigenetic marks, regulates cellular homeostasis. In mammalian cells DNA methylation mostly occurs at the 5’-position of cytosine within the CpG dinucleotide, which is not equally distributed along the genome, clustering in regions called CpG islands [15]. DNA methylation profiles show important differences among tissues [16] and several studies have reported a role for DNA methylation in cellular differentiation [17,18,19,20,21,22], including the intestine [23]. Pioneering investigations proposed a crypt niche model based on DNA methylation profiles [24] and, more recently, Kaaij et al [25] have shown an impact of transcription factor binding on shaping the DNA methylation landscape during differentiation of stem cells *in vivo*. Moreover, Sheaffer et al have demonstrated that dynamic DNA methylation at enhancers is required for stem cell differentiation in the small intestine [23].

The disruption of the epigenetic landscape is considered to be a common hallmark of many widespread diseases including cancer [26]. While tumor progression is characterized by global DNA hypomethylation [27] and hypermethylation of selected CpG islands (reviewed in [28,29]), most tissue specific DNA methylation changes occur in the lower CpG density regions that lie in close proximity of CpG island, known as CpG island shores [30]. It is well known that intestinal stem cell dynamics and premalignant disease is accompanied by DNA methylation changes [31], but it is yet unclear how the epigenetic control of cell differentiation is overridden in early stages of cell transformation [32].

Besides genome-wide DNA methylation profiles have been investigated in murine intestinal cell differentiation [23,25] and mouse intestinal adenomas [33], no study to date has addressed the concurrent and comparative analysis of both processes. We aim to identify the epigenetic determinants that set either the normal program of differentiation or the aberrant and progressive reprogramming into malignant cells. We have analyzed the DNA methylation profiles along the mouse small intestine crypt and early stages of tumorigenesis (*Apc* ^*Min/+*^ mice adenomas) using a sensitive and feasible genome-wide approach. Gene expression analyses were performed to determine the impact of DNA methylation in transcriptional regulation and the cellular expression signature.

## Materials and Methods

### Tissues and cell lines: ethical statement

All mice used were in a C57BL/6J background housed in centralized accredited research animal facilities, staffed with trained husbandry, technical, and veterinary personnel. Animal care, monitoring, and sacrifice were conducted in accordance with protocols approved by the Animal Care and Use Committees of the Barcelona Science Park (PCB) and the Barcelona Biomedical Research Park (PRBB), and the Ethical Committee for Animal Experimentation of the Government of Catalonia. Food and water were provided to the mice *ad libitum*. Ten adult wild type animals (>8 weeks old) were euthanized in a chamber with a saturated CO_2_ atmosphere. Intestines were extracted and cellular fractions from small intestine were obtained by purification of intestinal crypt cell based on the *EphB2* surface levels as described [7]. Four *Apc* ^*Min/+*^ mice were also used for this study. The general condition of *Apc* ^*Min/+*^ mice was monitored daily using animal fitness and weight controls throughout the experiment. When animal suffering was detected, they were administering with analgesics (Buprenorphine in a dose of 0.05–0.1 mg/kg of weight). When deteriorating clinical alterations were observed mice were euthanized in a chamber with a saturated CO_2_ atmosphere. Adenomas and normal colon tissue were obtained from *Apc* ^*Min/+*^ mice as previously described [34]. Mouse colon carcinoma cell line CT26 was obtained from the American Type Culture Collection (ATCC).

### Experimental design

**Fig 1:**
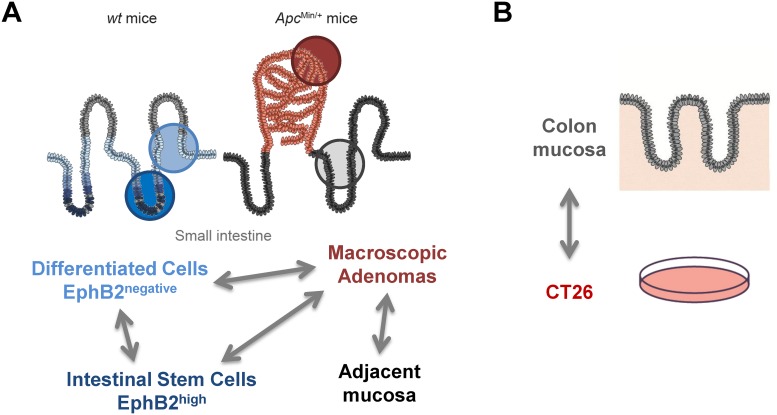
Simplified scheme of the experimental design to identify differential DNA methylation in intestinal cell differentiation and cancer. Setting **A** examines changes related with cell differentiation processes and early stages of intestinal cancer. Setting **B** allows the identification of differences between the large intestine and the CT26 colon cancer cell line representing advanced stages of cancer progression. Illustration of the intestinal epithelium is based on schemes published in reference [82].

Two experimental settings were designed for analysis of DNA methylation profiles (Fig 1). The first setting allowed the comparison of *EphB2* ^*high*^ small intestine stem cells (ISC) with the *EphB2* ^*negative*^ counterpart (differentiated cells) and adenomas and their adjacent non-tumor tissue from *Apc* ^*Min/+*^ mice. Intestinal crypts were sorted into four populations of cells representing different levels of differentiation based on the presence of *EphB2* (*EphB2* ^*high*^, *EphB2* ^*medium*^, *EphB2* ^*low*^ and *EphB2* ^*negative*^). The ISC gene expression signature [7] was analyzed to confirm the profile of the fractions and only the extreme fractions (*EphB2* ^*high*^, and *EphB2* ^*negative*^) were included in the study to avoid overlap among differentiation stages. In a second setting, we compared colon and small intestine mucosa from healthy mice and the CT26 cell line. All samples were analyzed by AIMS-Seq in duplicate. Validation of the DNA methylation and gene expression results were performed in additional samples obtained using the same procedures. The list of samples analyzed in this study and the molecular studies performed in each one are summarized in Table A in S1 File.

### AIMS-Seq

To compare the DNA methylation profiles at the genome scale we have applied next generation sequencing to the products of the Amplification of InterMethylated Sites technique (AIMS-Seq) (S1 Fig). AIMS allows the identification of differentially methylated DNA fragments flanked by two *XmaI* sites (CCCGGG) when two or more samples are compared [35]. AIMS principles and protocol have been described in detail elsewhere [35,36]. Briefly, genomic DNA (obtained by conventional phenol–chloroform extraction) was digested using the methylation-sensitive enzyme *Sma*I (Roche Diagnostics, GmbH, Mannheim, Germany) during 16h at 25°C, which generates blunt ends in the unmethylated sites. A second digestion with the methylation-insensitive isoschizomer *Xma*I (New England Biolabs, Boston, MA) was performed (6h at 37°C) generating cohesive ends in the methylated targets. The fragments flanked by cohesive ends (methylated) were selectively ligated to specific adaptors (5’CCGGTCAGAGCTTTGCGAAT and 5’CCGAATTCGCAAAGCTCTGA) using T4 DNA ligase (New England Biolabs, Boston, MA). Products were purified with Illustra DNA and Gel Band Purification Kit (GE Healthcare, Piscataway, NJ, USA). Fragments flanked by the adaptor were amplified by PCR with primer (ATTCGCAAAGCTCTGACCGGG). PCR product was purified with the JETQUICK PCR Spin Kit (Genomed, St. Louis, MO, USA), sheared with Covaris S2 system and the library was clustered and sequenced in Illumina GA2 devices. Reads (36 nucleotides long) were mapped with Bowtie [37] against the mouse genome build NCBI37/mm9. Only reads mapping uniquely inside virtual amplicons of the AIMS universe were taken into account for analysis of differential display. The AIMS virtual universe includes all the mouse genome fragments flanked by two XmaI sites and with a length range between 20 and 2000 bp. A total of 50,745 virtual amplicons were considered (Table B in S1 File). The number of reads mapping inside each amplicon represent an arbitrary indicator of methylation level of the affected XmaI sites. By comparing the number of reads mapped to a given amplicon in two different samples, it is possible to detect differential methylation: the sample with a higher number of reads will be more methylated in a given region. Total-sum scaling normalization was applied considering the number of reads mapping inside virtual amplicons (Table C in S1 File). Reproducibility assays were performed to test the variability of the technique. Due to limited availability of cells, AIMS-Seq was performed with 300 ng of genomic DNA for of intestinal crypt fractions and adenomas. Normal tissues, adenomas and cell lines were analyzed using 1000 ng of DNA. Results generated with different amounts of genomic DNA were not comparable due to the differential display of a large number of amplicons (data not shown).

Differentially methylated regions (DMRs) were revealed as differentially represented amplicons determined using the DESeq R package [38], considering significantly different those amplicons that had a log2 fold change value ≥3 and adjusted p-value ≤0.01. Differentially methylated domains (DMD) were identified using the circular binary segmentation method developed to analyze DNA copy number data [39]. Only genomic regions >50 kb and encompassing a minimum of five AIMS-Seq amplicons were considered. AIMS-Seq amplicons located in X and Y chromosomes were excluded in the global quantification of DMRs.

### Bisulfite treatment

Bisulfite treatment was performed using the EZ DNA methylation kitTM (Zymo Research). Specific genomic regions were amplified by nested PCR (primers are listed in Table D in S1 File) and the purified product was sequenced with the BigDye Terminator v3.1 Cycle Sequencing Kit (Applied Biosystems, Foster City, CA, USA).

### Gene expression

RNA was extracted with RNeasy Mini or RNeasy Micro Kit (Qiagen), according to manufacturer’s instructions and including the DNase digestion step. Labeled RNA was hybridized following one color protocol into SurePrint G3 Mouse 8x60K Agilent arrays. Data analysis was performed using the TMEV software applying Limma statistics (two class analysis) [40]. Gene expression differences with log2 fold change ≥1.2 and with a FDR ≤0.01 were considered significant.

### Genomic annotation and functional analysis

AIMS-Seq amplicons were annotated according to the overlap of the two flanking XmaI with selected genomic elements in the NCBI37/mm9 assembly. Genomic elements were downloaded from the mm9 annotation database at the UCSC browser (http://hgdownload.soe.ucsc.edu/goldenPath/mm9/database/).

Genes exhibiting changes at either DNA methylation or expression were analyzed for functional enrichment using the Functional Annotation Chart from DAVID Bioinformatics Resources 6.7 (http://david.abcc.ncifcrf.gov/) and taking into account the Gene Ontology classification (GO FAT category) and KEGG pathway [41]. FDR ≤0.05 was considered significant.

## Results

### Genome-wide analysis of DNA methylation by AIMS-Seq

AIMS-Seq generated an average of 14 million of reads uniquely mapped in the mouse genome (Table C in S1 File). Technical replicates were performed in samples pooled from several animals (intestinal crypts cell fractions) and biological replicates in tissues and cell lines (Table A in S1 File). Taking into account PCR limitations, only reads mapping inside canonical amplicons (ranging in size from 20 to 2,000 bp) were considered (S1B Fig). Noteworthy, 92–98% of all reads mapping unambiguously were located inside the AIMS-Seq virtual universe (Table C in S1 File).

**Fig 2:**
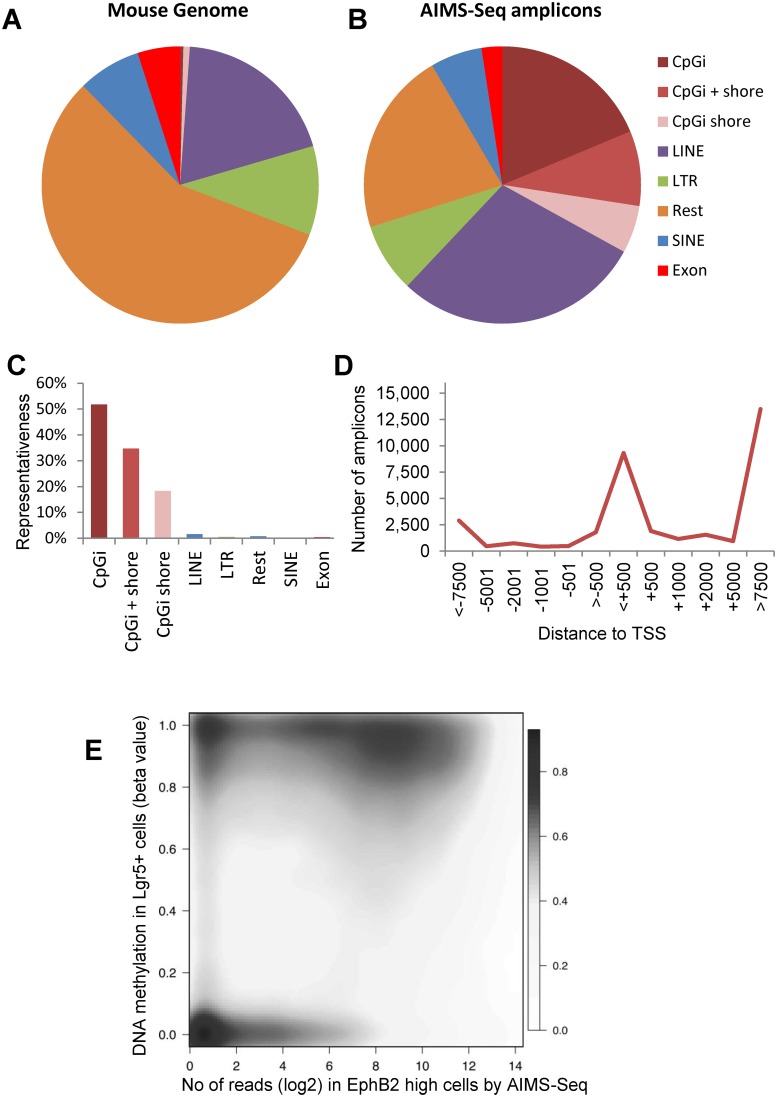
Genomic representativeness of AIMS-Seq. **A**, Mouse genome composition according to the different genetic elements considered. **B**, Relative distribution of AIMS-Seq amplicons in the mouse genome. **C**, Coverage of genetic elements by AIMS-Seq amplicons. **D**, Distribution of AIMS-Seq amplicons regarding its distance to the transcription start site (TSS) of the closest gene. **E**, Density distribution of AIMS-Seq amplicons according to the number of reads detected in ISCs compared with the beta values of the amplicon flanking CpGs determined in Lgr5+ cells analyzed by WGBS (data from reference [25]).

AIMS-Seq offers a broad representativeness of most types of genetic elements, although the amplicons are not evenly distributed among them (Table B in S1 File). Therefore we analyzed the genomic features of regions represented in the AIMS-Seq virtual universe against the whole genome (Table B in S1 File). The adscription of the functional and structural features was based on the location of the XmaI sites flanking each amplicon. As compared with the relative contribution of the different genetic elements to the mouse genome (Fig 2A), AIMS-Seq amplicons are especially enriched in CpG islands, exons and 5’UTR and depleted in non-coding RNAs and SINEs (Fig 2B and Table B In S1 File). It is of note that a large proportion of CpG islands and the neighboring regions (CpG island shores) are represented in AIMS-Seq (Fig 2C). Indeed, the density of amplicons within 1kb from transcription start sites is also very high (Fig 2D). A comparison with data generated by WGBS in a similar setting [25] revealed that AIMS-Seq amplicons represented with a high number of reads tend to be flanked by methylated CpGs, while fully unmethylated CpGs are poorly amplified (Fig 2E).

### Differential DNA methylation along intestinal cell differentiation and tumor progression

The number of reads assigned to each canonical amplicon was normalized according to the total number of reads mapping unambiguously in the mouse genome (Table C in S1 File) and used as an index of DNA methylation for sample comparisons. Differentially methylated regions (DMRs) in cell differentiation along the small intestine crypt and early states of tumorigenesis were determined using the DESeq package (see Material and methods).

**Fig 3:**
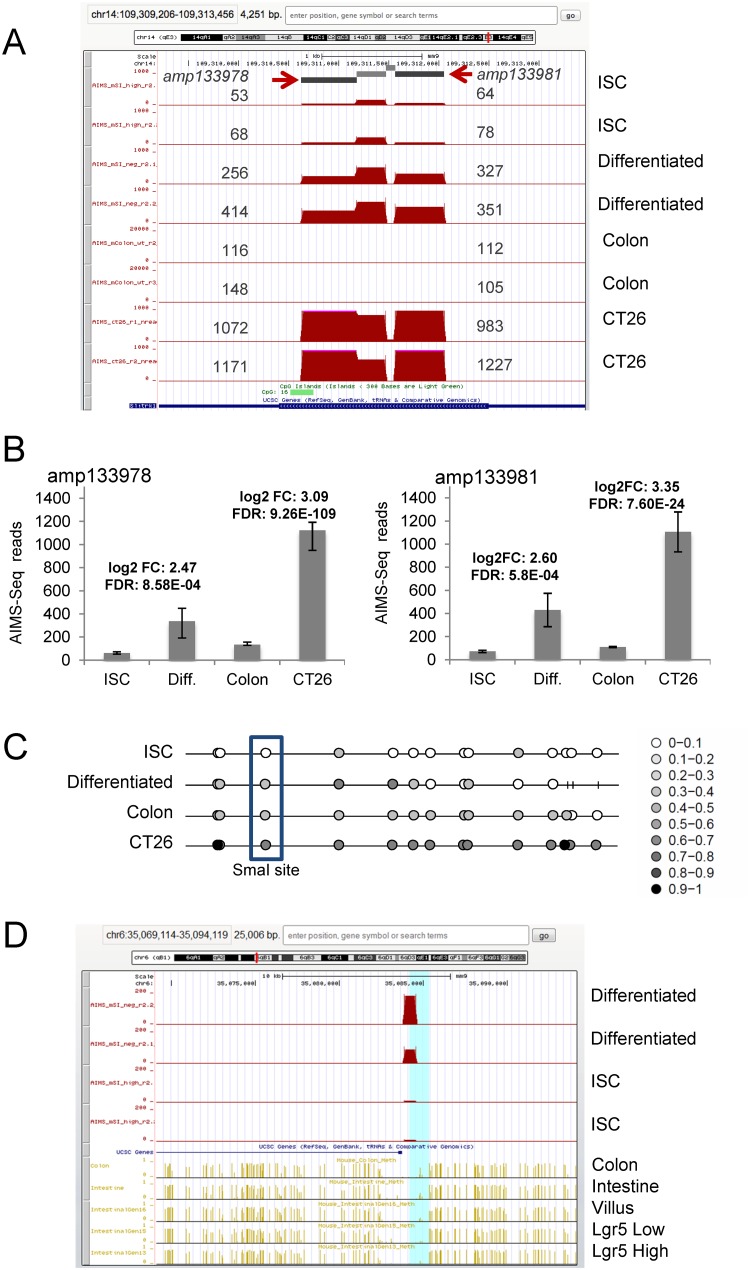
AIMS-Seq analysis. **A**, Distribution of normalized reads obtained by AIMS-Seq in the genomic region neighboring the Slitrk1 gene. Amplicons amp133978 and amp133981 are indicated by arrows and the number of reads mapped in each one is shown next to the peak in four types of cells (ISC, differentiated, colon and CT26 cells) analyzed in duplicate. **B**, Both amplicons showed statistically significant differences according to the defined criteria (see Material and methods) in the comparison ISC vs. Differentiated cells and normal colon vs. CT26 cells. The logarithm of the fold change (log2FC) and the false discovery rate (FDR) are indicated for two comparisons between samples. **C**, Methylation profile of CpGs surrounding the XmaI site of amp133978 in four samples. The profile was represented using the Methylation plotter tool [83] and the methylation level of each CpG (beta value) is represented by a grey scale lollipop from white (unmethylated) to black (fully methylated) according to the scale. Sticks represent CpGs not determined. The CpG corresponding to the 3’ *Xma*I site is boxed. **D**, Comparison of the differential methylation detected by AIMS-Seq in ISC and differentiated cells with data generated by WGBS in related samples. Note the progressive increase of methylation from intestinal stem cells (Lgr5+) to more differentiated states. WGBS data were obtained from references [25,42].

Validation studies by direct bisulfite sequencing were applied to a subset of the DMRs detected by AIMS-Seq. Illustrative examples are shown in Fig 3 and S2 Fig. It is of note that most differences detected by AIMS-Seq in the cell differentiation process corresponded to moderate changes in the levels of DNA methylation. In some cases, the methylation change only affected part of the amplicon. This may be the case of *XmaI* sites located in the boundaries of certain genomic elements as CpG islands or CpG island shores.

**Fig 4:**
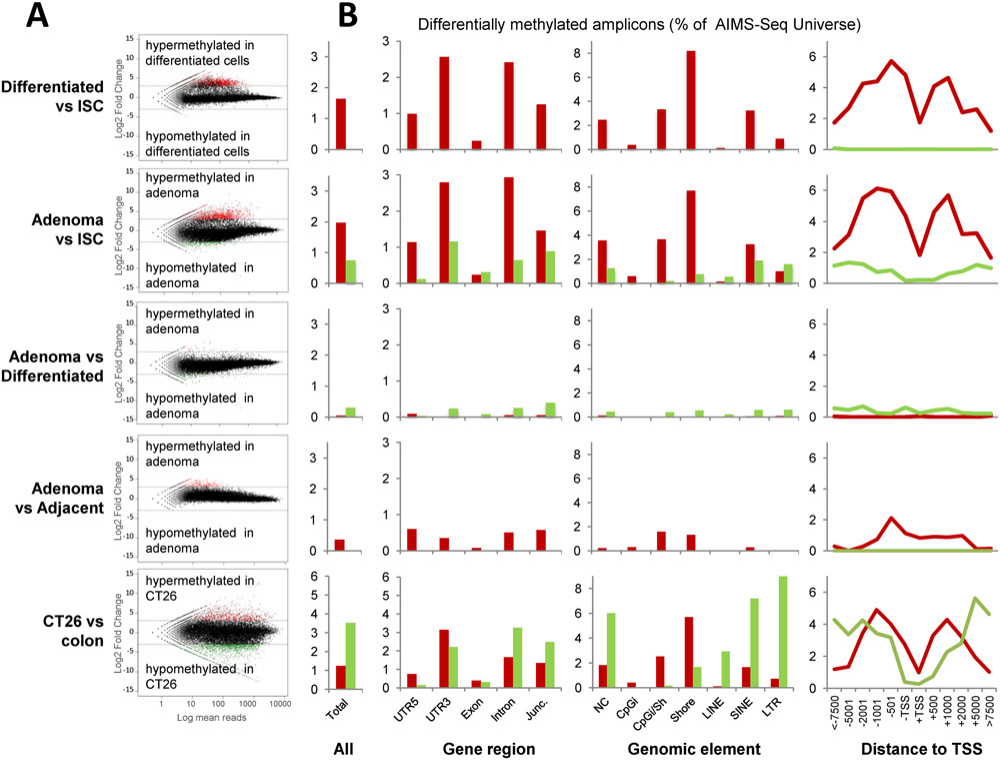
Distribution of differentially methylated AIMS-Seq amplicons. **A**, The results of five different comparisons (as illustrated in Fig 1) are distributed in rows. MA plots show the log2 ratio of the number of normalized reads per amplicon in the indicated comparisons. **B**, Relative distribution (% of the AIMS-Seq virtual universe) of hypermethylations (red) and hypomethylations according to different genomic features regarding gene context (UTR, exons, introns, junction exons/introns), genome element (CpG island, CpG island shore, LINE, SINE, LTR and non classified elements) and distance to transcription start site (TSS).

At the global level, only 1.6% of AIMS-Seq amplicons displayed changes in small intestine differentiated cells versus intestinal stem cells, with an overwhelming prevalence of hypermethylations (n = 831) versus hypomethylations (n = 5) (Table 1 and Fig 4). In adenomas, 2.7% of amplicons were found differentially methylated when compared with intestinal stem cells. Interestingly, hypermethylations represented the most abundant changes in adenomas and a large proportion of them were shared with differentiated cells (Table 1). About 2/3 of the hypermethylations within 2kb of the TSS were shared by differentiated cells and adenomas (S3 Fig), which indicates they are not specific of the transformation process. This hypothesis is also supported by the lower overlap (about 1/5) with the hypermethylations detected in CT26 cells. Nevertheless, it remains to be elucidated whether they are required for cell transformation. Alternatively, hypomethylations in adenomas versus ISCs were observed to a lesser extent than hypermethylations (Fig 4); and unlike hypermethylations, they appeared to be specific of the tumor process, because they were absent in the cell differentiation process. When compared with differentiated cells (from healthy animals) and adjacent tissue (from *Apc* ^*Min/+*^ mice), adenomas showed a low number of DMRs with opposite trends: prevalence of hypomethylations respect normal differentiated cells but hypermethylations versus the adjacent tissue (Table 1).

**Table 1:** Differentially methylated regions (DMR) as determined by AIMS-Seq during intestinal cell differentiation and transformation.

	Hypermethylated		Hypomethylated	
Comparison	no of amplicons	% of AIMS-Seq	no of amplicons	% of AIMS-Seq
Differentiated *vs*. ISC	831	1.64	5	0.01
Adenomas *vs*. ISC	993	1.95	297	0.58
Adenomas *vs*. differentiated	15	0.03	96	0.19
Adenoma vs. Adjacent	178	0.35	0	0.00
CT26 vs. colon	628	1.24	1,655	3.26

Finally, the colon carcinoma cell line CT26, representing an advanced stage of the tumor process, presented alterations in 4.5% of amplicons with a predominance of hypomethylations (Table 1 and Fig 4).

### Genetic features of differentially methylated loci

Regarding the genomic distribution of DMRs, the proportion of both hypermethylations and hypomethylations in different genetic elements was quite different depending on the comparisons considered (Fig 4 and S2 Fig). Results are summarized in Tables E to I in S1 File and the lists of DMRs together with the most relevant biological information are reported in Tables J thru R in S1 File.

Gains of methylation, as the most prominent trend in intestinal cell differentiation, affected 3’UTRs, introns, CpG island shores, non coding RNAs and frequently occurred in the proximities of TSSs but excluding the TSS itself (Fig 4). Hypomethylations affected all genomic elements with no specific enrichment (Fig 4). Moreover, hypermethylations exhibited by the adenomas affected CpG island shores and 5’UTRs of genes.

CT26 cells, representing advanced stages of carcinogenesis, show a higher number of DNA methylation changes in which hypermethylations follow genomic profiles (3’UTRs, lincRNAs and CpG island shores) similar to cell differentiation and early cell transformation processes, whereas hypomethylations show a broader genomic impact, being more prominent in gene desert regions and repetitive sequences (Fig 4 and Tables E thru I in S1 File).

### Functional impact of differentially methylated genes during intestinal differentiation and cancer

To gain insights into the biological implications of DNA methylation changes we analyzed functional enrichment of genes with DMRs within 2Kb of the TSS (Tables J-R in S1 File). Hypomethylations were not enriched in any specific functional category in any of the comparisons performed. On the other hand, developmental and regulatory processes were enriched in the hypermethylated compartment of differentiated cells and adenomas as compared with ISCs (Table S in S1 File). Interestingly, in addition to these categories, CT26 cells also exhibited significant enrichment of hypermethylation in genes related with cell migration and adhesion (Table S in S1 File), which are critical to convey invasive features to malignant cells.

Genome-wide analysis of DNA methylation in multiple mouse tissues has shown that most tissue specific DMRs occur at distal *cis*-regulatory elements [42]. Therefore we analyzed the distribution of DMRs detected by AIMS-Seq in regard to the localization of predicted enhancers as reported in adult mouse intestine [42]. Interestingly, both cell differentiation and malignant transformation showed enrichment of hypermethylations in the vicinity or overlapping with predicted enhancers, whereas hypomethylations only affected enhancer regions during the differentiation process (S4 Fig).

### Chromosomal domains with coordinated differential methylation during intestinal cell differentiation and cancer

**Fig 5:**
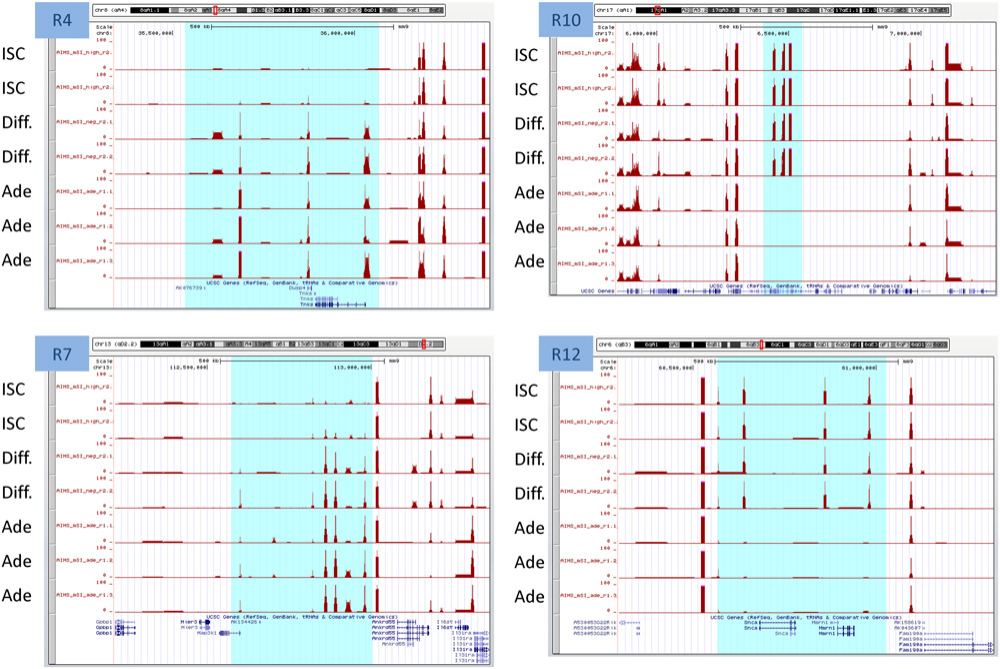
Differentially methylated domains in Differentiated-ISC and Adenoma-ISC comparisons. Four examples are illustrated. Each track corresponds to the number of normalized reads in the AIMS-seq for each sample. Highlighted regions indicate domains larger than 50 kb and with at least 5 amplicons showing differential methylation between samples. R4 and R7 domains are hypermethylated in differentiated and adenoma cells versus ISCs. R10 and R12 domains are hypomethylated in adenoma cells versus ISCs and differentiated cells.

To determine the occurrence of regional changes in the DNA methylation profiles during the cell differentiation and transformation processes, we compared the differential representation of AIMS-Seq reads along the mouse genome by applying CGH-like analysis (see Material and methods). Twenty-two differentially methylated domains (DMD) were identified among all comparisons performed (Permutation test with 100 permutations, p-value = 0.01). Only regions represented by 5 or more AIMS-Seq amplicons and affecting >50 Kb were considered (Table T in S1 File). Four of these regions were reproduced in more than one comparison. Interestingly, differentiated cells exhibited 8 DMDs ranging from 80 Kb to 2.58 Mb in length, all of them hypermethylated when compared with ISCs. A 67 Kb region in chromosome 13 including *Tmem181b-ps* and Gm2792 pseudogenes was hypomethylated in adenomas (in comparison with differentiated and ISC), but hypermethylated in CT26. The large 2.58 Mb region hypermethylated in differentiated cells as compared with ISC included a subregion of 882 Kb that was also hypermethylated in adenomas (Fig 5). This region contains 109 genes, including two gene families: there are two clusters of histone coding genes at the 5’ and 3’ ends, while in the middle there is a vomeronasal type-1 receptor family cluster (Table T in S1 File). The detected increase of methylation was mostly located in the region occupied by the histone coding genes, which is a CpG rich region. Functional analysis showed enrichment for nuclear and chromosome organization, largely explained by the presence of multiples isoforms of histone 1 (Table U in S1 File).

### Differential gene expression along intestinal cell differentiation and tumor progression

**Fig 6:**
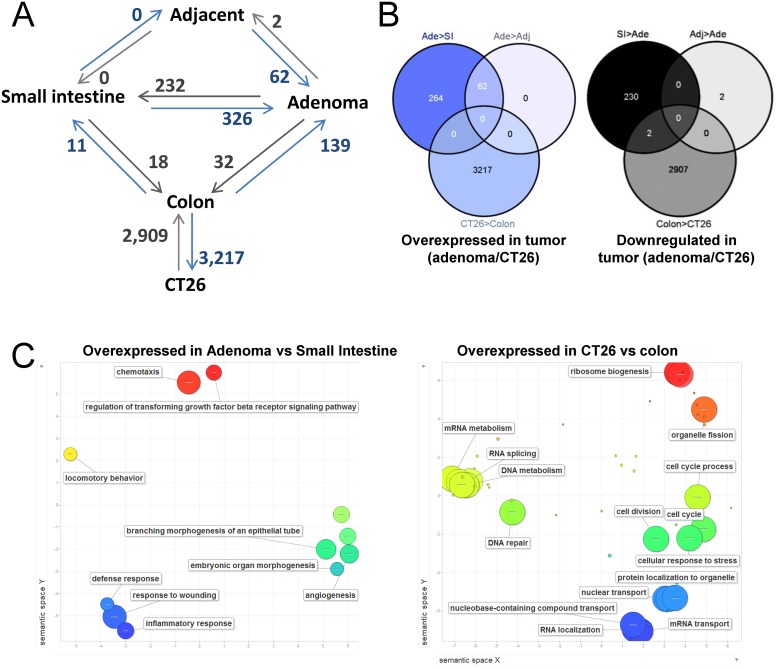
Differential gene expression. **A**, Diagram of the gene expression analyses performed by microarrays. Comparisons are denoted by connecting lines. The number of genes overexpressed in the sample pointed by the arrowhead is indicated next to the connecting line. **B**, Overlapping of genes differentially expressed in different pair of samples. **C**, Semantic representation of biological processes enriched in genes overexpressed in adenomas and CT26 cells compared with the respective normal tissue. The graph was generated with Revigo [84]. Bubble size indicates the-log10 of the FDR (see Table K in S2 File for details).

In addition to DNA methylation, we also compared the gene expression signatures among healthy small intestine and colon, *Apc* ^*Min/+*^ mouse adenomas and their adjacent non-tumor tissue, and CT26 cell line. Genes with differential expression are described in detail in Tables A to J in S2 File and a summary of the differences is illustrated in Fig 6. Normal tissues (Small intestine and colon of control animals versus normal tissue adjacent to adenomas in *Apc* ^*Min/+*^ mice) showed few changes: 29 genes were differentially expressed between small intestine and colon, whereas non tumor tissue adjacent to adenomas in *Apc* ^*Min/+*^ mice was indistinguishable from the small intestine of healthy animals. Interestingly, of the 326 genes overexpressed in adenomas as compared with healthy small intestine, only 62 were overexpressed when the comparison was performed against the tissue adjacent to the adenoma, indicating that the adjacent tissue displays intermediate levels for about 81% of genes overexpressed in adenomas (Fig 6). The cellular functions of genes overexpressed in adenomas showed enrichment in chemotaxis, angiogenesis and regulation of transforming growth factor beta-receptor signaling pathway (Fig 6C and Table K in S2 File). Unlike adenomas, CT26 cells exhibited a high number of differences with normal colon and with similar rates of over- and downregulation (Fig 6 and Tables A to J in S2 File). As expected, multiple biological processes related with cancer were affected (Fig 6C and Table K in S2 File).

### Lack of global correlation between differential methylation and gene expression

Next, we investigated the correlation between DNA methylation changes and gene expression data generated in another study [7]. By AIMS-Seq we determined that in differentiated cells, 397 had a gain of methylation in the promoter region (2kb from the TSS) (Table L in S2 File). Most genes did not show any association between DNA methylation and gene expression changes (data not shown). Only 14 genes exhibited inverse correlation between methylation and expression (Table M in S2 File). Strikingly, ten of these hypermethylated AIMS-Seq amplicons were located in the gene promoter region of the downregulated genes (*Aqp4*, *Axin2*, *Atad5*, *Brca1*, *Fads1*, *Lgr5*, *Phgdh*, *Slc1a2*, *Slit2*, *St3gal3*) belonging to the expression signature of the intestinal stem cell [7], which represents a highly significant enrichment (Fisher exact test, p value <2.2e-16). These changes affected the gene CpG island and/or CpG island shore, except for *Aqp4*. Direct bisulfite sequencing (S2 Fig) and inspection of WGBS profiles obtained by Kaaij et al in an independent setting (S5 Fig) revealed that the changes corresponded to moderate variations in the percentage of DNA methylation in one or several CpG sites.

Genes becoming hypermethylated in CT26 are low expressed in healthy colon when compared with genes maintaining methylation levels constant or hypomethylated (S6 Fig). Indeed, a global trend was observed in hypermethylated genes that showed even lower values in CT26 cells (S6 Fig).

## Discussion

### Genome-wide analysis of differential DNA methylation, technical remarks

Our knowledge of biological processes as cell differentiation and cancer has improved dramatically in the last two decades thanks to the application of advanced genomic tools to decipher the epigenetic code. Besides this progress and the wide range of available methodologies [43,44,45], genome scale analysis of DNA methylation dynamics still represents critical technical challenges.

The use of methylation sensitive endonucleases played a pivotal role in the early characterization of genomic regions affected by DNA methylation and the discovery of disease specific epigenetic biomarkers [43,44]. With the advent of next generation sequencing technologies, the potential of techniques based in this type of approaches has reemerged. Methyl-Seq is one of such examples developed in Richard Myers lab and applied to identify DNA methylation changes associated with human liver development [46].

Here we have applied AIMS-Seq, a variation of the AIMS method [35]. This technique requires minute amounts of material (<500 ng of genomic DNA) and has a very high sensitivity, which makes it adequate for the experimental setting described here. The standard version of AIMS has been applied with a significant success to uncover specific [47,48,49] and global DNA methylation alterations in cancer [50,51,52], epigenetic differences between monozygotic twins [53] and was instrumental for the characterization of functional CpG methylation in the honeybee [54].

The coupling of high throughput sequencing to AIMS (AIMS-Seq) renders an approach based in the same principles of Methyl-Seq and allows the study of differential DNA methylation at the genome scale level, but at a relatively low cost. AIMS-Seq targets about half of the sites covered by Methyl-Seq, but this reduction of complexity results in a five-fold increase in read depth per amplicon with a similar sequencing effort. AIMS-Seq reports information on a wide range of sequences, including those with low CpG content, which have been described as the most variable regions in healthy tissue [55] and repetitive sequences, which are poorly analyzed by other approaches. About half of the CpG islands and CpG island shores in the mouse genome get canonical representation (Fig 2). Another important advantage of AIMS-Seq is the simplicity of data analysis, as currently available bioinformatic and biostatistical pipelines can be applied with minimal adaptations, overcoming the computational bottleneck that often slows down this type of projects.

A limitation of enzyme-based methods to detect differential DNA methylation is that they rely in a single CpG site. Nevertheless, in a recent study using whole genome bisulfite sequencing data we have shown that scoring of DNA methylation in a single CpG is highly representative of the status of a whole CpG island [56]. This is also the case for other genomic elements (Jordà M et al, manuscript in preparation). Therefore, the target of the methylation sensitive restriction enzymes can be used as a faithful surrogate indicator of the methylation status of the comprising genomic element.

An important feature of this AIMS-Seq is its high sensitivity to detect differential methylation affecting a small percentage of cells [35]. It should be noted that a large proportion of the changes observed in the DNA methylation levels in similar settings by WGBS are below 10–20% [25,42,57], and AIMS-Seq is able to detect these small variations (S5 Fig). These features make AIMS-Seq a feasible approach to identify new epigenetic targets in settings where DNA methylation differences are small and sample availability is limited. The suitability of AIMS-Seq in this type of studies has been well exemplified by its application to analyze DNA methylation profiles in murine myogenesis [58]. Carrió et al. uncovered more than 1000 differentially methylated regions providing an overall view of the epigenetic landscape along the muscle-lineage determination process. Indeed, the comparison with data generated by Reduced Representation Bisulphite Sequencing allowed a global validation of the results [58]. This study also demonstrated the adequacy of AIMS-Seq to identify specific changes with a prominent role in the cell differentiation process. In particular, they found hypomethylation of Myf5 superenhancer (a crucial myogenic regulatory factor) as a key epigenetic event associated with the functional activation of this locus [58].

In summary AIMS-Seq provides an overall view of the differential methylome between multiple biological conditions and allows the identification of specific candidates with a potential role in the process. Most outstanding features AIMS-Seq are high sensitivity, low material requirements and a relatively small demand of technical and computational labors.

### DNA methylation dynamics in intestinal differentiation

Our analysis indicates that intestinal cell differentiation is accompanied by small changes in DNA methylation, mostly consisting in hypermethylations that affect less than 2% of the genome analyzed by AIMS-Seq. As a whole, these figures are difficult to compare with WGBS data generated in similar experimental settings [23,25]. Kaaij et al reported 297 TSS with >50% DNA methylation difference and most of them corresponded to hypomethylations in differentiated cells as compared with ISC. For their sake, Sheaffer et al identified 4240 DMRs and more than half of them affected CpG islands and CpG island shores. In our setting, about 1/3 of hypermethylations occurring during cell differentiation were located near TSS (less than 500 bp).

DNA methylation changes in enhancers play an important role in cell identity and differentiation processes (reviewed in reference [59]); and previous investigations have reported that a large proportion of DMRs affect enhancers during cell differentiation [23,25,60]. Our results are in agreement with these observations, but also expand the scope of the conclusions by showing that hypermethylations also affect distal regulatory elements in mouse intestinal tumors, as previously shown in different human cancer types [61,62,63]. However, unlike cell differentiation, our data indicate the spreading out of hypomethylation to non-regulatory regions in intestinal tumorigenesis. It is well known that the functional implications of global genomic hypomethylation in cancer go beyond the deregulation of specific genes (reviewed in reference [26,27])

Only a small proportion of these epigenetic changes were associated with transcriptional silencing of the respective gene (Table M in S2 File). Similar studies applying WGBS [23,25], which offers a more comprehensive genomic coverage, have found good correlation between hypermethylations near TSSs and gene expression. The overall lack of correlation between DNA methylation and gene expression in our study may be explained by the high sensitivity of AIMS-Seq to detect DMRs. Direct comparison with WGBS data [25] revealed that most hypermethylations detected by AIMS-Seq were very small and affected a few CpG sites. Interestingly, genes reported to constitute the ISC signature (and therefore downregulated or silenced in differentiated cells, i.e. *Lgr5*) showed very small increases of DNA methylation in a few CpGs in the respective CpG island shore (S5 Fig). By AIMS-Seq these changes were clearly detected, which confirms the sensitivity of this technique.

A note of caution should be sounded regarding the interpretation of DNA methylation changes detected in intestinal cell differentiation. Due to the small amounts of starting material, fractions from four animals were pooled in order to reach enough DNA to perform the analyses. This implies that inter-animal variability was abolished from our data. While this limitation does not challenge our results and conclusions, we cannot fully disregard inter-individual variability, as well as heterogeneity in analyzed cell fractions. Our data on specific changes with a role in the intestinal cell differentiation (e.g.: *Lgr5* hypermethylation, see above) have been confirmed by ourselves and other studies [23], but further studies using more powerful techniques are needed to determine the steadiness of epigenetic and transcriptional profiles in intestinal cell differentiation.

### DNA methylation dynamics in intestinal tumorigenesis

*Apc* ^*Min/+*^ adenomas displayed few changes in DNA methylation when compared with intestinal stem cells (2.7% of the canonical AIMS-Seq amplicons), and a large subset of them were shared with differentiated cells (in the case of hypermethylations), and the non-tumor tissue adjacent to the adenoma (in the case of hypomethylations). It has been previously reported that the dynamics of hypermethylation and hypomethylation are independent in human colorectal cancer [51,64,65]. In this context, we can postulate that hypomethylation would be an early phenomenon preceding the morphological emergence of the adenoma and consistent with a field effect [66] in which the adjacent tissue already displays a large fraction of the molecular changes bear by the tumor. Moreover, hypermethylation in adenomas would recapitulate in part the ISCs differentiation process and incorporate tumor specific changes. The conservation of DNA methylation profiles may explain the retention of cell differentiation capacity in *Apc* ^*Min/+*^ microadenomas [67]. In fact most *Apc* ^*Min/+*^ adenomas maintain the crypt architecture and do not progress to carcinoma [68]. The subsequent cell transformation would require additional changes consistent with the stepwise model of cancer progression in which genomic and epigenomic instability drive subsequent alterations.

Recently, Grimm et al. [33] applied MeDIP-seq to study the methylome of *Apc* ^*Min/+*^ adenomas. Similarly to our analysis, a high proportion of DMRs affected CpG islands and promoter regions. Indeed, they found polycomb targets were enriched among hypermethylated regions in adenomas, although just a few tumor suppressor genes were silenced. Binding of Polycomb repressive complex 2 (PRC2) has been shown to point genes prone to be hypermethylated in cancer [69]. Our data confirm Grimm’s observations, as 64 of the genes found hypermethylated in adenomas and 76 in CT26 by AIMS-Seq constitute targets of the PRC2 complex [70] (S3B Fig). The enrichment of PRC2 targets in the DMRs detected by AIMS-Seq is highly significant (Fisher exact test, p value <2.2e-16). This group of genes includes the homeobox gene *En1* (S7 Fig), whose hypermethylation has been described as a promising biomarker of neoplastic stages of human colorectal cancer [71].

### Hypermethylation affects large chromosomal domains in cell differentiation and cancer

We have identified several regions displaying coordinated hypermethylation of all the encompassed amplicons. Interestingly, differentiated cells and adenomas shared many of the hypermethylated domains. The nature and functional implications of DMDs are unknown. Nevertheless, it is tempting to compare hypermethylated DMDs with the well characterized Long Range Epigenetic Silencing (LRES) phenomenon initially described in human colorectal cancer [48] and also found in murine tumors [72]. LRES appears as the concurrent hypermethylation of multiple CpG islands and downregulation of most of the genes in a genomic region ranging from hundreds of kilobases to a few megabases [73]. Although LRES was discovered with the classical version of the AIMS method, our current data are insufficient to assimilate the differentially hypermethylated domains identified here to LRES. It should be noted that AIMS-Seq is not the most appropriate method to detect epigenetic changes affecting large chromosomal regions due to the interspersed nature of its genomic coverage. Specific regional analyses are required to clarify the nature of DMDs as it was done for LRES in different settings [48,71,72,74,75,76]. On the other hand, genome-wide approaches have identified large chromosomal domains undergoing extensive DNA hypomethylation in human colorectal cancer [64,65]. In our setting, hypomethylation was relatively scarce and no large domains were detected. This may be due to the technical reasons, as the AIMS-Seq coverage is high in gene-rich regions and near the promoters (Fig 1 and reference [35]), but low in gene poor regions and nuclear lamina associated domains where most of the hypomethylation occurs [64,65].

### Concluding remarks

In summary, the AIMS-Seq approach appears as a sensitive and feasible tool to compare the epigenetic profiles of samples with very limited availability. Our analysis shows that DNA methylation changes along small intestine crypt differentiation and early stages of tumorigenesis are few and small compared with late stages. Hypermethylations constitute the predominant change and are enriched in genomic regions related with gene regulation. Both processes exhibit highly overlapping panels of genes with DNA methylation changes; besides tumor related hypermethylation appears as a progressive phenomenon reaching deeper methylation levels in advanced stages. On the other hand, hypomethylation shows different signatures in differentiation and cancer and is already present in the non-tumor tissue adjacent to the adenomas in *Apc* ^*Min/+*^ mice, which is consistent with a role in the promotion of tumorigenesis [77,78].

Our findings may be also relevant to the comprehension of these processes and the underlying epigenetic mechanisms in the human counterpart. Different studies have pinpointed the parallelisms between mouse and human colorectal cancer at epigenetic level [33,72,79] and many of the genes we have found hypermethylated in early stages of mouse tumorigenesis (i.e., *Lgr5*, *Axin2* and *En1*) may have potential clinical applications in human cancer [71,80,81].

## Supporting Information

S1 Fig Features of AIMS-Seq technique. **A**, Scheme of the AIMS-Seq technique. **B**, Representation of the read coverage against amplicon length. To facilitate visualization, only 1,000 out of 50,745 amplicons have been plotted. Left panel shows the mean values obtained for the first set of samples (Fig 1A); right panel shows the results for the second set (Fig 1B).
(TIF)

S2 Fig Bisulfite sequencing validation of differentially represented AIMS-Seq amplicons. Data for eight amplicons is shown. Genomic localization of the amplicons is shown. Graphs on the left show the number of normalized AIMS-Seq reads in each sample. The amplicon ID is shown on top f each graph. Logarithmic fold change values in base = 2 (log2FC) and False Discovery Rate (FDR) are indicated for each comparison (colon vs Ct26 and ISC vs differentiated cells). The results obtained with direct bisulfite sequencing are represented as lollipops using the Methylation plotter tool (Mallona et al, 2014, reference [79]). Each circle represents a CpG dinucleotide and the degree of grey indicates the methylation level from fully methylated (black) to completely unmethylated (white). Sticks represent CpG sites not determined. Blue boxes identify XmaI sites corresponding to amplicon flanks. Colon: normal colon mucosa, ISC = Intestinal Stem Cell, Diff = Differentiated cells, CT26 colon cancer cell line. Data may be obtained from Tables J through R in S1 File.
(TIF)

S3 Fig Differentially methylated genes. **A**, Overlap of hypermethylated genes as detected by AIMS-Seq. Only genes with one or more AIMS-Seq amplicons within 2Kb of the Transcription Start Site (TSS) are considered. **B**, Distribution of hypermethylated genes in adenomas (versus ISC) and CT26 cells (versus normal colon) and PRC2 targets.
(TIF)

S4 Fig Features of differentially methylated amplicons. **A**, Density plot of AIMS-Seq amplicons according to their distance (log10 of bp) to the nearest enhancer region. Red line shows the distribution of all amplicons included in the virtual AIMS-seq universe. Black line shows the distribution of DMRs for each comparison as indicated in the graph. Statistical significance (p-value) for distribution of enhancers inside/outside DMRs, within 1Kb of the DMRs, and the Kolmogrov-Smirnov (KS) test are shown on top of each graph. **B**, Summary of the results: Enhancer associated hypermethylations and hypomethylations are enriched in the indicated comparisons.
(TIF)

S5 Fig UCSC Genome Browser (NCBI37/mm9) representation of AIMS-Seq reads in two genomic regions displaying DNA methylation changes detected by AIMS-Seq (highlighted area) and associated with silencing of the respective gene (*Lgr5* and *St3gal3*) during the differentiation process of ISCs. Whole genome bisulfite sequencing data from Kaaij et al. [25] is shown for comparison. Note small changes in DNA methylation levels in the boundaries of the amplicon.
(TIF)

S6 Fig Differential DNA methylation and gene expression levels. Genes were classified according to its differential DNA methylation between CT26 and healthy colon tissue as determined by AIMS-seq amplicons as follows: constant (no gains or losses of DNA methylation), hypermethylated and hypomethylated. Only genes with one or more AIMS-Seq amplicons within 2Kb of their Transcription Start Site were considered. CT26 hypermethylated genes showed a statistically significant downregulation in CT26 (Wilcoxon test, p<2.2e-16). No differences were observed for constant or hypomethylated genes.
(TIF)

S7 Fig Differential DNA methylation of En1 CpG island shore detected by AIMS-Seq in intestinal cancer. Each track indicates the number of reads mapped in AIMS-Seq amplicons.
(TIF)

S1 File Table A, Samples used in the study and analyses performed with each sample. Table B, Distribution of virtual AIMS-Seq amplicons in the mouse genome (mm9). Table C, Distribution of AIMS-Seq reads. Table D, Primer sequences. Table E, Differentially methylated AIMS-Seq amplicons, by genomic context. Table F, Differentially methylated AIMS-Seq amplicons, by gene context. Table G, Differentially methylated AIMS-Seq amplicons, by transcript type. Table H, Differentially methylated AIMS-Seq amplicons, by genomic element. Table, Differentially methylated AIMS-Seq amplicons, by distance to TSS. Table J, Hypermethylated in Differentiated vs ISC. Table K, Hypermethylated in Adenoma vs ISCs. Table L, Hypermethylated in Adenoma vs Differentiated. Table M, Hypermethylated in Adenoma vs Adjacent. Table N, Hypermethylated in CT26 vs Colon. Table O, Hypomethylated in Differentiated vs ISC. Table P, Hypomethylated in Adenoma vs ISCs. Table Q, Hypomethylated in Adenoma vs Differentiated cells. Table R, Hypomethylated in CT26 vs Colon. Table S, Functional enrichment of differentially methylated AIMS-Seq amplicons near TSS Genes. Table T, Differentially methylated domains (DMD). Table U, Functional enrichment in DMD R6.1.
(XLSX)

S2 File Table A, Differential gene expression. Table B, Differential gene expression. Table C, Differential gene expression. Table D in S2 File. Differential gene expression. Table E, Differential gene expression. Table F, Differential gene expression. Table G, Differential gene expression. Table H, Differential gene expression. Table I, Differential gene expression. Table J, Differential gene expression. Table K, Functional enrichment in gene differential expression in intestinal cell differentiation and cancer. Table L, Gene expression/DNA methylation correlations. Table M, Hypermethylated regions associated with gene downregulation in Differentiated versus ISC.
(XLSX)
