# Supplementary figures and images for: Overlapping DNA Methylation Dynamics in Mouse Intestinal Cell Differentiation and Early Stages of Malignant Progression
Source: PLoS One. 2015 May 1;10(5):e0123263. doi: 10.1371/journal.pone.0123263 (PMC4416816; doi:10.1371/journal.pone.0123263)

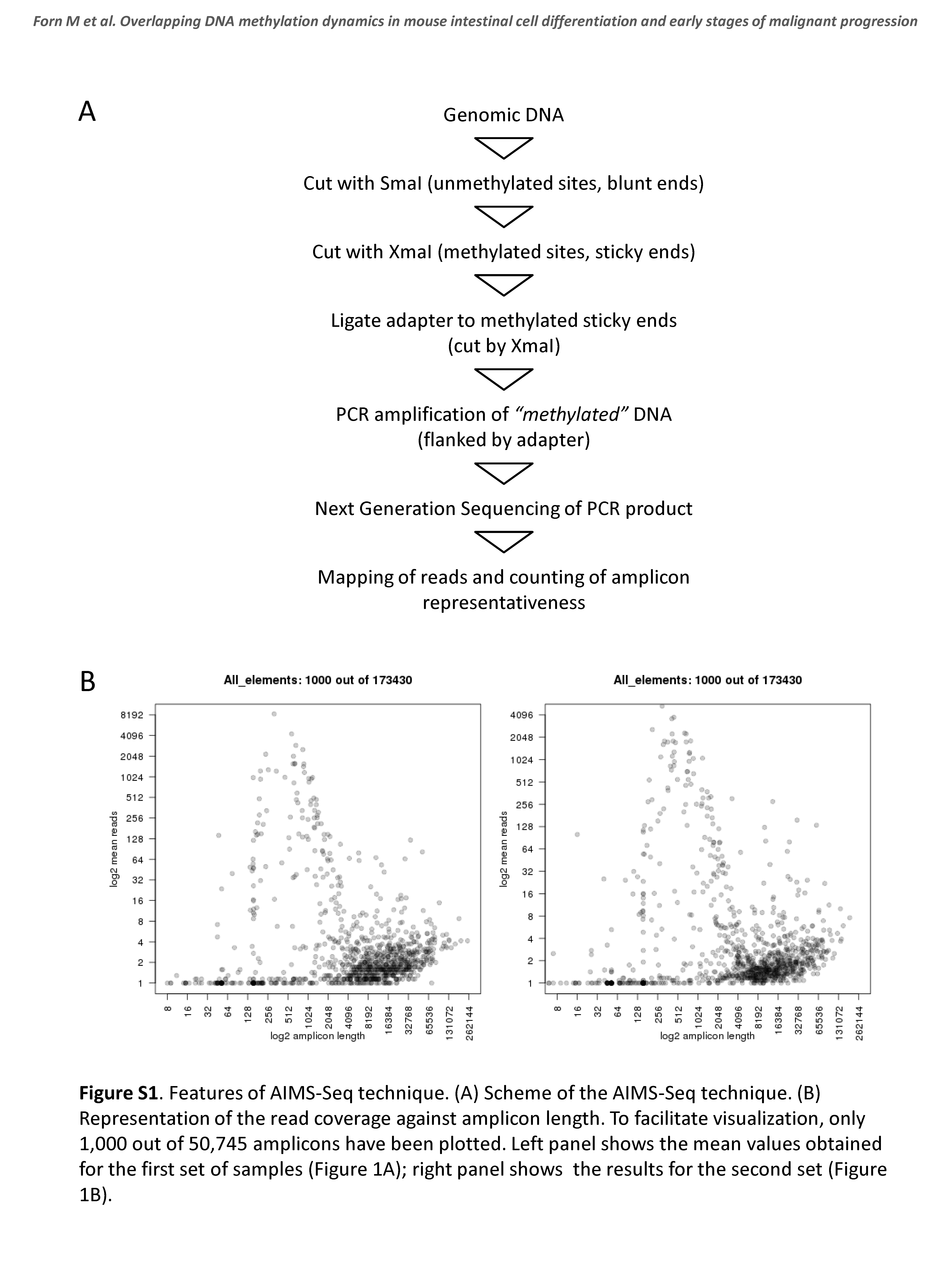

Supplement: S1 Fig — A, Scheme of the AIMS-Seq technique. B, Representation of the read coverage against amplicon length. To facilitate visualization, only 1,000 out of 50,745 amplicons have been plotted. Left panel shows the mean values obtained for the first set of samples (Fig 1A); right panel shows the results for the second set (Fig 1B). (TIF) [file pone.0123263.s001.tif]

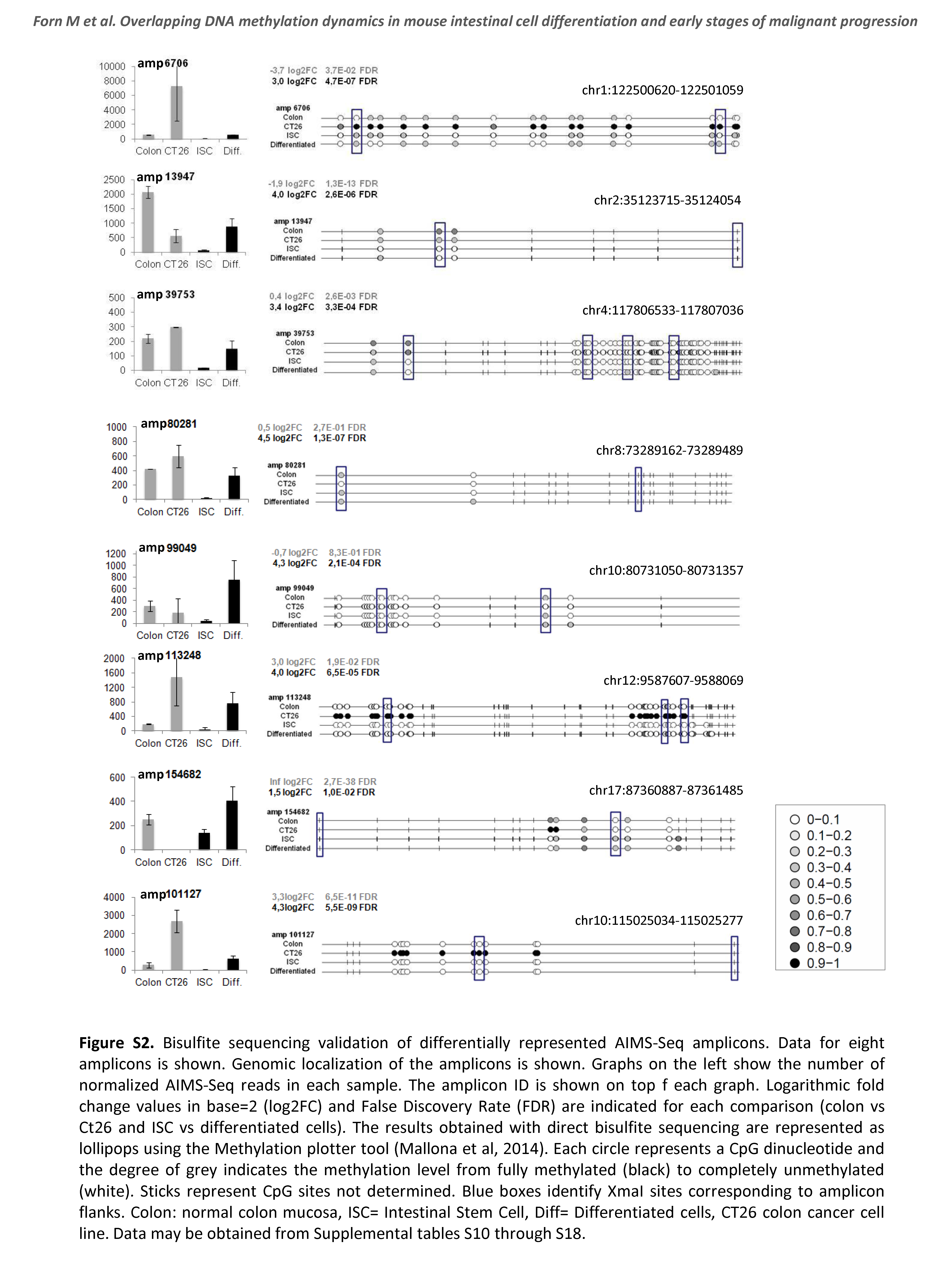

Supplement: S2 Fig — Data for eight amplicons is shown. Genomic localization of the amplicons is shown. Graphs on the left show the number of normalized AIMS-Seq reads in each sample. The amplicon ID is shown on top f each graph. Logarithmic fold change values in base = 2 (log2FC) and False Discovery Rate (FDR) are indicated for each comparison (colon vs Ct26 and ISC vs differentiated cells). The results obtained with direct bisulfite sequencing are represented as lollipops using the Methylation plotter tool (Mallona et al, 2014, reference [79]). Each circle represents a CpG dinucleotide and the degree of grey indicates the methylation level from fully methylated (black) to completely unmethylated (white). Sticks represent CpG sites not determined. Blue boxes identify XmaI sites corresponding to amplicon flanks. Colon: normal colon mucosa, ISC = Intestinal Stem Cell, Diff = Differentiated cells, CT26 colon cancer cell line. Data may be obtained from Tables J through R in S1 File. (TIF) [file pone.0123263.s002.tif]

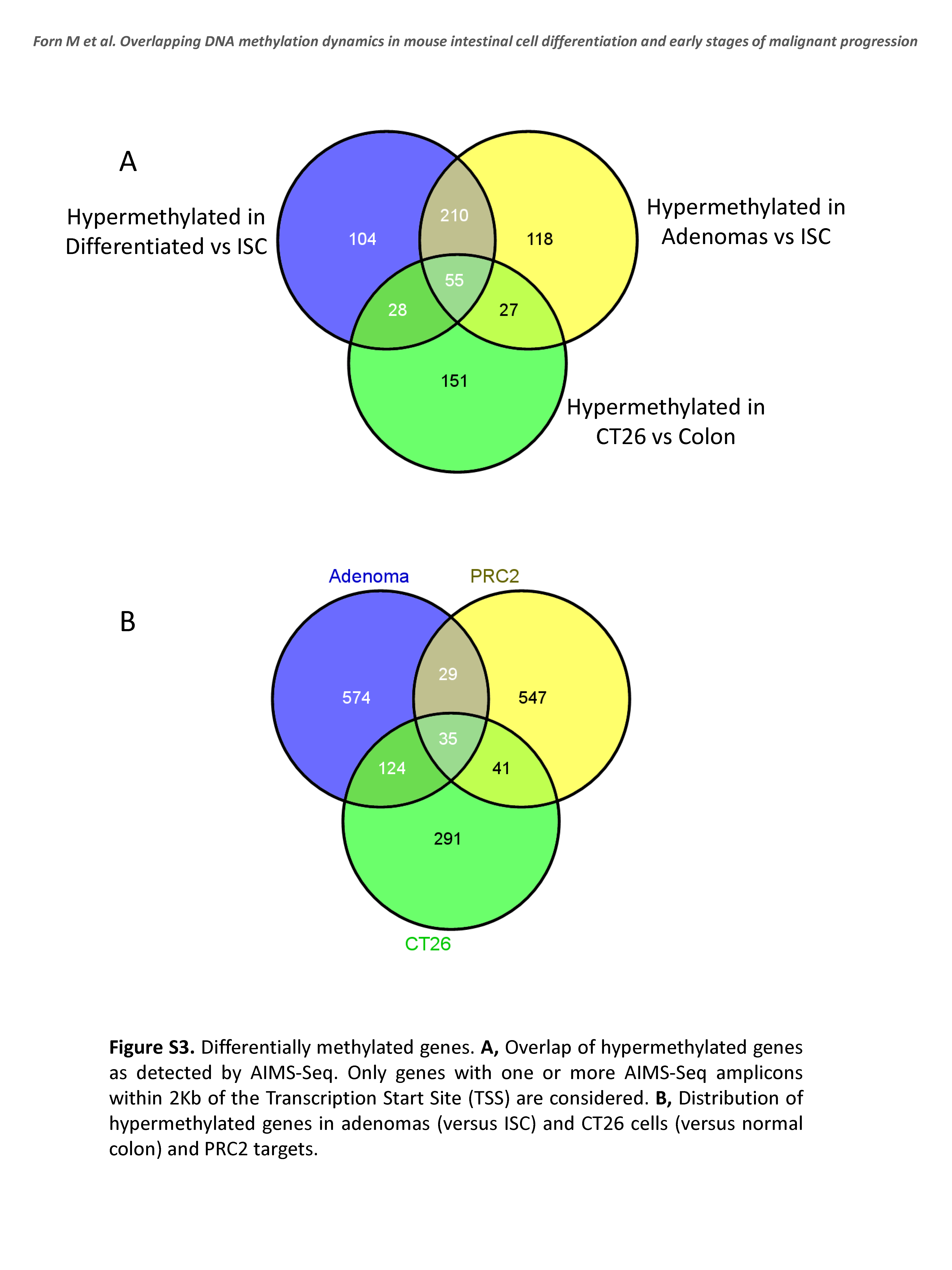

Supplement: S3 Fig — A, Overlap of hypermethylated genes as detected by AIMS-Seq. Only genes with one or more AIMS-Seq amplicons within 2Kb of the Transcription Start Site (TSS) are considered. B, Distribution of hypermethylated genes in adenomas (versus ISC) and CT26 cells (versus normal colon) and PRC2 targets. (TIF) [file pone.0123263.s003.tif]

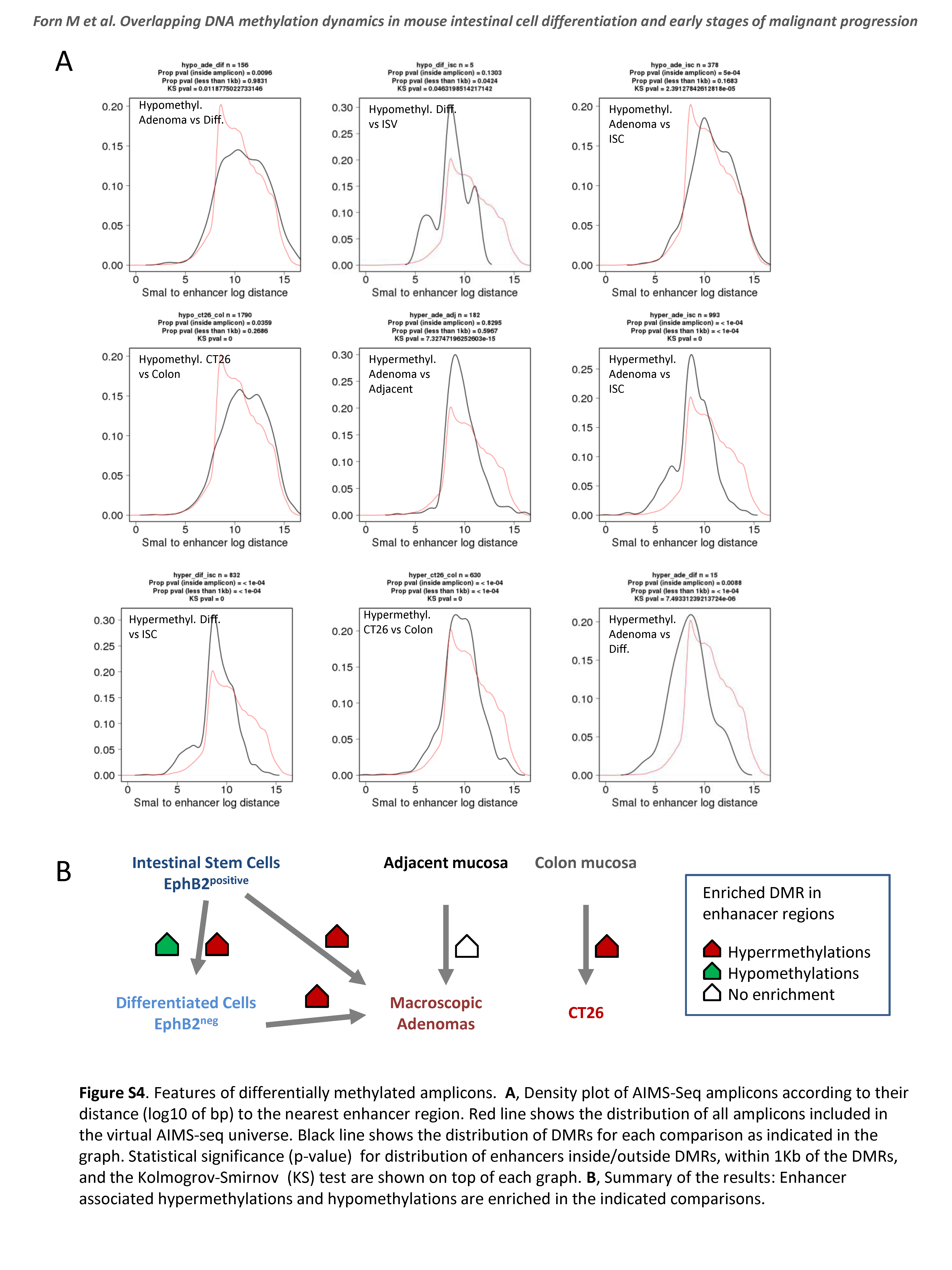

Supplement: S4 Fig — A, Density plot of AIMS-Seq amplicons according to their distance (log10 of bp) to the nearest enhancer region. Red line shows the distribution of all amplicons included in the virtual AIMS-seq universe. Black line shows the distribution of DMRs for each comparison as indicated in the graph. Statistical significance (p-value) for distribution of enhancers inside/outside DMRs, within 1Kb of the DMRs, and the Kolmogrov-Smirnov (KS) test are shown on top of each graph. B, Summary of the results: Enhancer associated hypermethylations and hypomethylations are enriched in the indicated comparisons. (TIF) [file pone.0123263.s004.tif]

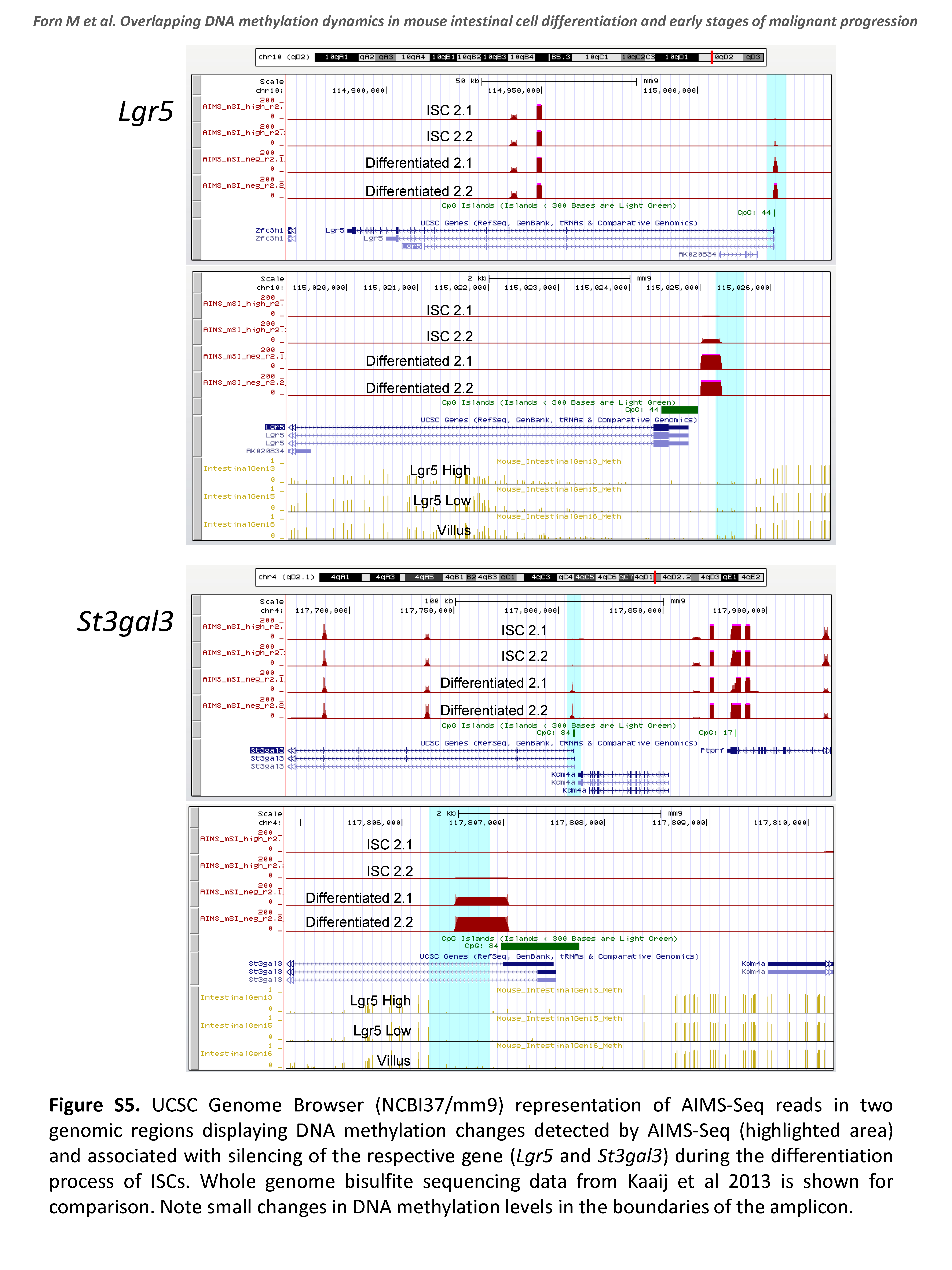

Supplement: S5 Fig — Whole genome bisulfite sequencing data from Kaaij et al. [25] is shown for comparison. Note small changes in DNA methylation levels in the boundaries of the amplicon. (TIF) [file pone.0123263.s005.tif]

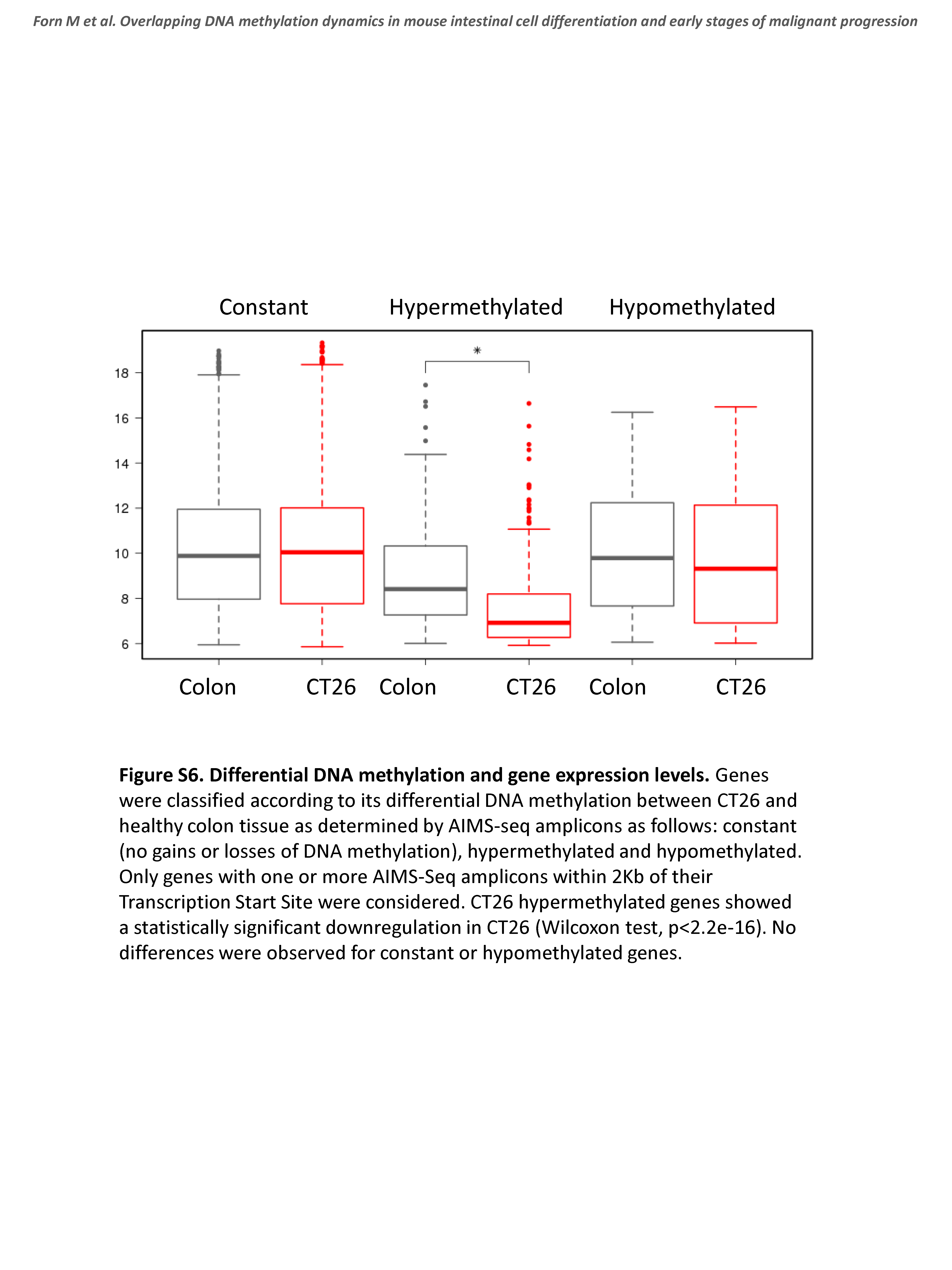

Supplement: S6 Fig — Genes were classified according to its differential DNA methylation between CT26 and healthy colon tissue as determined by AIMS-seq amplicons as follows: constant (no gains or losses of DNA methylation), hypermethylated and hypomethylated. Only genes with one or more AIMS-Seq amplicons within 2Kb of their Transcription Start Site were considered. CT26 hypermethylated genes showed a statistically significant downregulation in CT26 (Wilcoxon test, p<2.2e-16). No differences were observed for constant or hypomethylated genes. (TIF) [file pone.0123263.s006.tif]

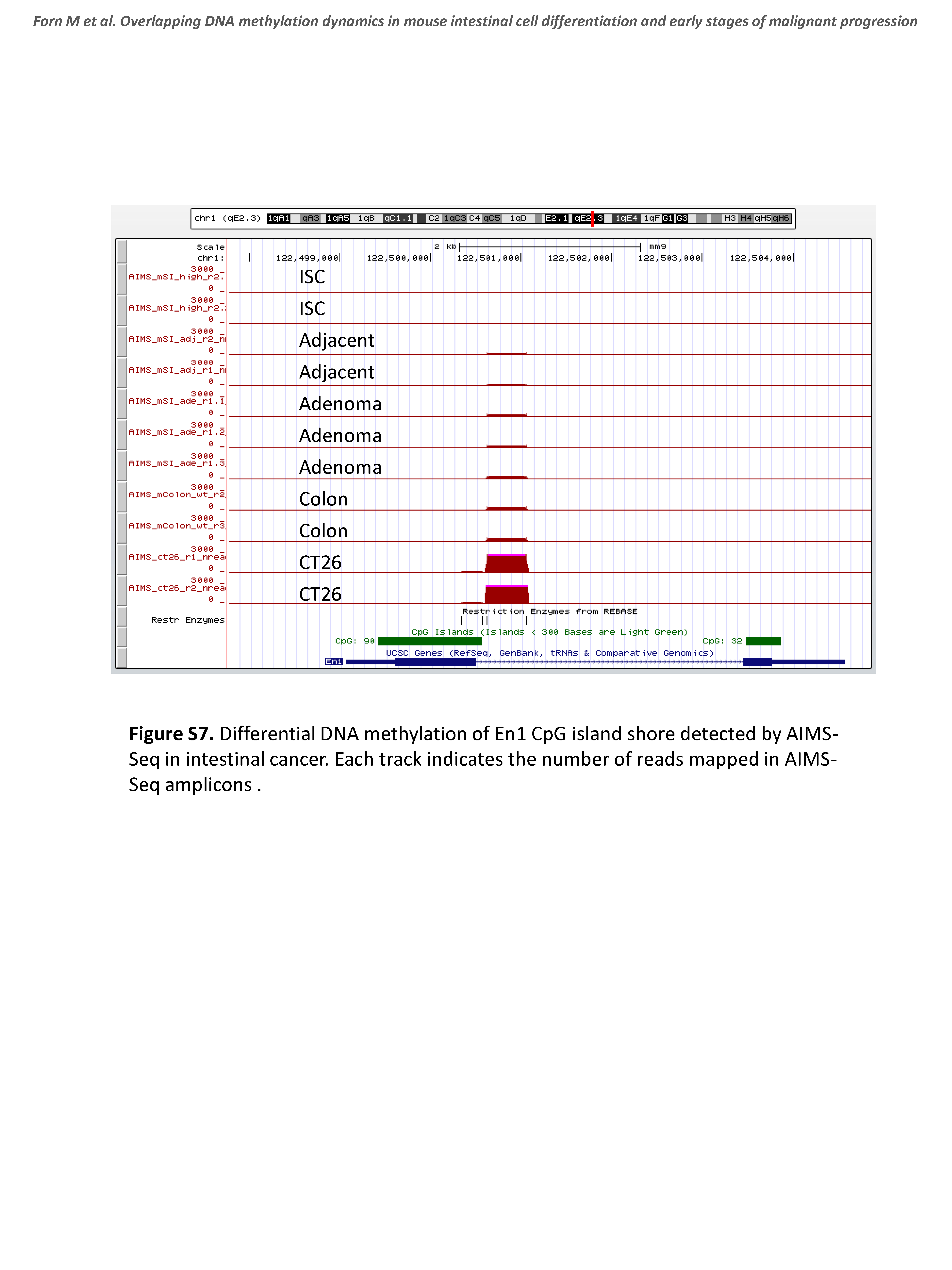

Supplement: S7 Fig — Each track indicates the number of reads mapped in AIMS-Seq amplicons. (TIF) [file pone.0123263.s007.tif]
